# L-lysine dietary supplementation for childhood and adolescent growth: Promises and precautions

**DOI:** 10.1016/j.jare.2024.05.014

**Published:** 2024-05-11

**Authors:** Rasika Gunarathne, Xiao Guan, Tao Feng, Yu Zhao, Jun Lu

**Affiliations:** a Auckland Bioengineering Institute, the University of Auckland, Auckland 1142, New Zealand; b School of Health Science and Engineering, University of Shanghai for Science and Technology, Shanghai 200093, China; c School of Perfume and Aroma Technology, Shanghai Institute of Technology, Shanghai 201400, China; d School of Life Sciences, Shanghai Normal University, Shanghai 200042, China; e Maurice Wilkins Centre for Biodiscovery, Auckland, New Zealand; f Department of Food and Agriculture Technology, Yangtze Delta Region Institute of Tsinghua University, Zhejiang, Jiaxing 314006, China

**Keywords:** Lysine supplement, Protein quality, Growth, Children and adolescents, Cereal diets

## Abstract

**Background:**

L-lysine (lysine) is an essential amino acid that plays a vital role in human nutrition. It serves as a key component in protein synthesis and fulfills critical roles in various physiological activities. For decades, lysine supplements have been extensively used to promote the growth and development of children, particularly in developing countries where cereal-based diets are everyday staples.

**Aim of the review:**

This review aims to provide an overview of the overall effectiveness of lysine supplements concerning the growth of children and adolescents. Additionally, it addresses the potential precautions that should be considered when using lysine supplements in this context.

**Key scientific concepts of review:**

Receiving lysine oral supplements and lysine-fortified cereal diets were observed to enhance nitrogen retention and improve anthropometric measurements such as height, weight, Z-scores, body mass index, and skinfold thickness. Furthermore, lysine positively influenced the children's developmental quotient and various serological biochemical parameters, such as hormones, immunological indicators, proteins, bone metabolic indicators, and red blood cell parameters. These supplements are generally considered clinically safe, with no reported toxicity where the related side effects are limited to subjective gastrointestinal tract symptoms. It is essential to be cautious about excessive intake of lysine, as it can lead to an imbalance of amino acids, thereby potentially suppressing its intended benefits. When used with appropriate precautions, lysine can serve as a safe supplement with promising benefits for the growth of children and adolescents. Nevertheless, further contemporary research studies on lysine supplementation would be insightful and valuable in better understanding its optimal use, potential benefits, and safety in promoting growth.

## Highlights

• This review provides an overview of lysine supplementation on the growth of children and adolescents.
• Consumption of lysine-supplemented diets improves nitrogen retention, anthropometric measurements, and biochemical parameters.
• Lysine supplementation shows significance in improving the nutritional quality of cereal-based diets.
• The use of optimal levels of lysine supplementation is essential to obtain the intended benefits.
• More comprehensive studies are needed to provide further insights into optimal and safe lysine supplement dosage.

## Graphical abstract

**Figure:**
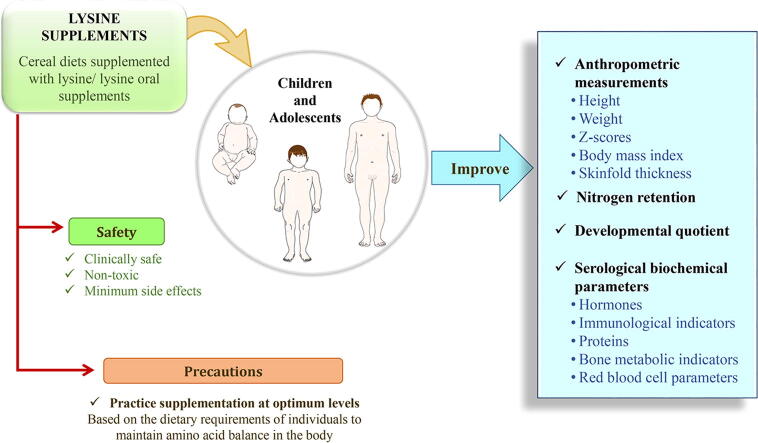

## Introduction

Amino acids are the fundamental units of proteins and play critical roles in various physiological activities of the human body, such as protein synthesis, body development, metabolism, neurotransmission, and maintaining osmotic pressure stability [1]. They are categorized as essential and non-essential, where the essential amino acids are the type that cannot be synthesized within the human body and thus need to be supplied to the body from exogenous sources to keep up the process of protein biosynthesis [2]. Limitations of any individual amino acid can interfere with whole protein synthesis, as the presence of all amino acids in adequate quantities in the human body is crucial for this process [3]. In recent times, there has been a significant rise in the intake of specific amino acids as dietary supplements, given their potential health benefits across both physical and psychological applications [4].

Lysine is an essential amino acid that is important for the nutrition and development of humans. It carries out a necessary role in protein synthesis as a predominant building block of the majority of proteins [5]. Therefore, it is compulsory to acquire lysine in adequate amounts through diet or supplements [6]. There is serious concern regarding adequate lysine intake through diet as it presents in limited quantities in many essential food sources like grains [3], thereby leading to a significant impact on the protein quality of cereal/pulse-based diets [7]. Additionally, the reactive nature of lysine causes it to be involved in many chemical reactions. As a result, a fraction of lysine in food systems can be lost by degradation and can become unavailable by interacting with other molecules. It is, therefore important to supply intact forms of lysine into the diet to overcome this collateral loss of biotemplate [8]. Consequently, individuals across different age groups may need to use dietary supplements to meet the recommended intake of lysine, recognizing the varying dietary requirements throughout different stages of life.

Lysine is actively involved in a wide range of essential physiological processes within the human body. While its primary function involves participating in protein synthesis [8], it also serves as a precursor for vital molecules like carnitine [9]. Furthermore, lysine supports bone health, boosts the immune system, aids in hormone production, and contributes to brain development [5], [7]. The requirement for lysine in children and adolescents has been internationally prioritized as it is fundamental for proper development, bone growth, and disease prevention. Several studies have been conducted to establish minimum dietary requirement values of lysine in children and adolescents. Notably, the minimum requirement was found to vary based on the age, nutritional, and health status of the tested populations [10], [11], [12], [13]. Responsively, World Health Organization (WHO) recommendations have specified lysine requirements for different age groups of infants, children, and adolescents.

Nevertheless, meeting these established lysine requirements remains uncertain in such multi-variable circumstances, as there is a high risk of insufficient dietary intake for the population, especially in developing nations. Insufficiency primarily results from factors such as reduced food consumption, reliance on cereals as a staple diet, and losses of lysine during food processing [7]. The lack of intake of this essential amino acid is reported as causative for a greater risk of a range of adverse medical conditions in children and adolescents. With the protein quality of a diet known to influence children's linear growth, insufficient amounts of lysine impair the overall protein quality and, consequently, have a negative impact on growth. Therefore, there is a potential risk for the children of developing countries who consume grain-based diets as their staple food to receive inadequate amounts of available lysine compared to the established daily intake levels [12]. In an attempt to overcome this issue, lysine is widely used as a nutritional supplement to improve the quality of diet [8].

In 1889, lysine was first isolated from casein and was introduced to the U.S. market in 1955 for the first time in the form of lysine hydrochloride [5], [6]. Over the past two decades, the global market for lysine has experienced remarkable growth, expanding nearly twentyfold, with worldwide production of lysine salts exceeding 2.2 million tonnes on an annual basis 5]. According to the facts, lysine hydrochloride was reported as a well-tolerated and safe dietary supplement for humans up to a dose of 3.0 g per day [14]. When going through the literature, it became evident that a significant number of studies on lysine supplements targeting children and adolescents have been conducted over the past few decades. Indeed, the findings of these studies have contributed substantially to understanding lysine supplementation, providing fundamental knowledge and insights on this aspect.

There is a pressing need for in-depth discussion on this aspect, given the widespread use of lysine supplements and fortified foods, as well as the lack of reviews examining the promising effects of lysine supplements on the nutritional and health status of children and adolescents. Therefore, this review aims to provide a comprehensive insight into the impact of lysine supplementation on the growth of children and adolescents. Further, it focuses on elaborating on the optimal levels of lysine supplementation, potential safety concerns, and precautions. This review will provide essential knowledge to understand lysine supplements and will encourage further extensive research on this aspect.

### The role of lysine in human body functions

Lysine has an integral role in the development and growth of the human body. The primary role of lysine in the human body is to participate in protein synthesis [8], [15]. It is also an essential compound for building a positive nitrogen balance in the body [3]. In addition, it promotes overall bone health by decreasing urinary calcium content, increasing calcium absorption, enhancing bone strength, and stimulating the activity of bone-forming cells [7]. Lysine is also a precursor of carnitine, which is a crucial molecule in fatty acid metabolism and energy production [9].

**Fig. 1:**
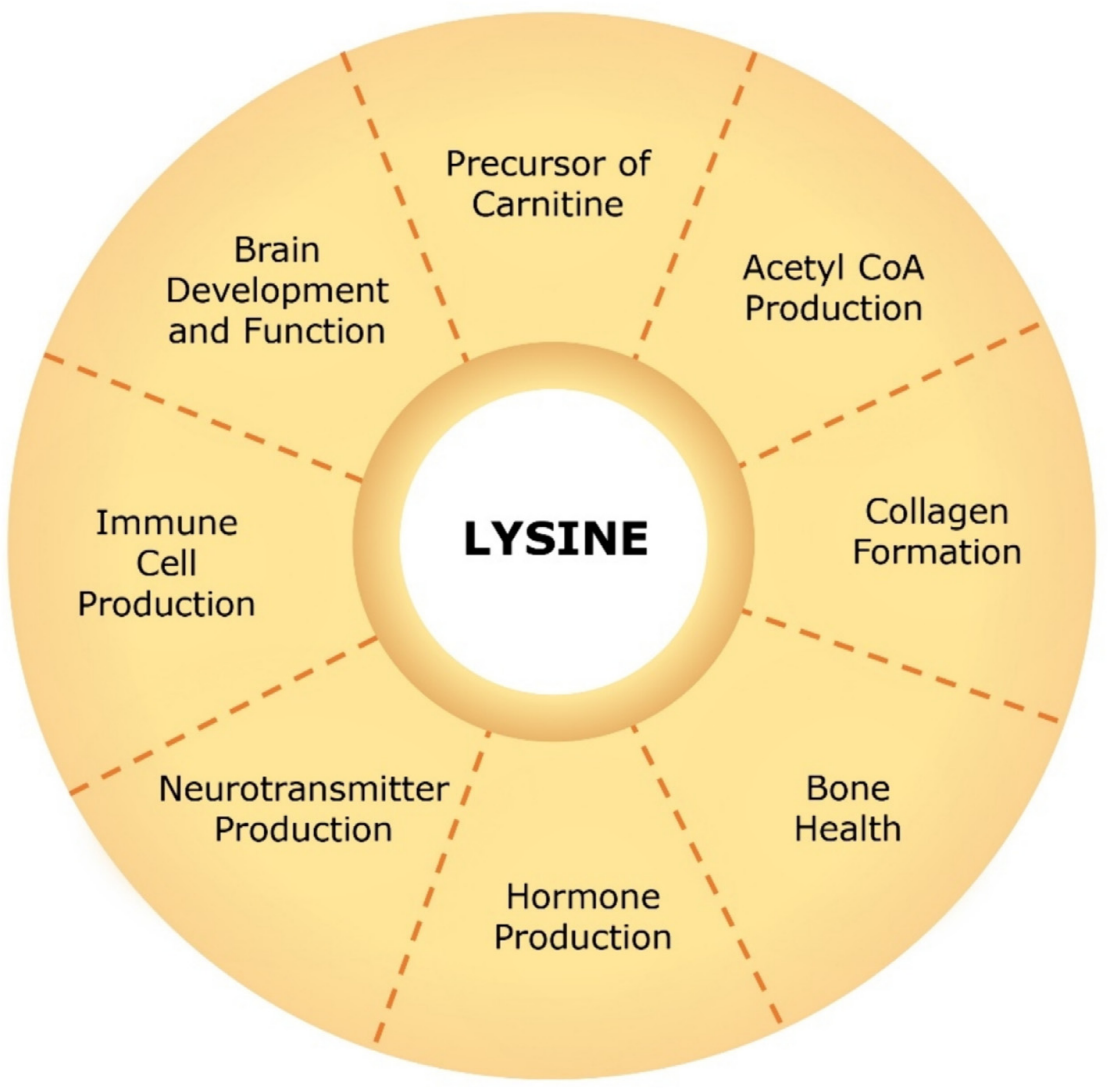
Role of lysine in human nutrition.

Lysine is involved in energy production via carbohydrate metabolism and the generation of acetyl CoA [7]. It plays a significant role in forming collagen, acting in tissue repair, and producing enzymes, antibodies, and hormones [5]. Lysine positively impacts both brain development and function and possesses neuroprotective properties in cases of cerebral ischemic injury [16] and intracerebral hemorrhage injury [17]. The prominent functions of lysine in the human body are illustrated in Fig. 1.

### The dietary requirement of lysine

Numerous studies have investigated the lysine requirement of children and adolescents in different age groups. WHO specifications for lysine in infants, children, and adolescents are also available, ranging from 30-60 mg/kg per day, with a decrease observed as the subjects age. The age group and the respective lysine requirements are shown in Table 1. Previously, the mean lysine requirements of enteral-fed term neonates were investigated, revealing that the established requirement stands at 130 mg/kg per day [13]. In this study, the lysine requirement of twenty-one subjects was analyzed employing the indicator amino acid oxidation (IAAO) method. Similarly, the mean lysine requirement of eleven postsurgical neonates receiving parental nutrition was estimated as 104.9 mg/kg per day, adopting the same IAAO method with modifications [18]_._ However, Snyderman et al. [10] reported that the lysine requirement of infants of postnatal ages (1–5 months) ranged from 90 to 105 mg/kg per day, employing the nitrogen-balance method for their determinations.

**Table 1:** The lysine requirement of infants, children, and adolescents.

	Protein (g/kg per day)		
Age (years)	Growth	Maintenance	Lysine requirement^1^ (mg/kg per day)
0.5	0.46	0.686	64
1–4	0.19	0.686	45
4–10	0.06	0.686	35
10–14	0.07	0.686	36
14–18	0.04	0.686	34
>18	−	0.66	30

1 Sum of the lysine requirement for maintenance (maintenance protein × the adult scoring pattern) and growth (tissue deposition adjusted for a 58 % dietary efficiency of utilization × the AA tissue pattern).

Lysine requirement of school children with different health statuses from various regions was investigated previously by adopting the IAAO method. For instance, a study on healthy school children in Canada revealed that the lysine requirement of the tested children was 35 mg/kg per day, and the population-safe lysine requirement was 58 mg/kg per day [11]. Values obtained elsewhere for Western children were found to be similar to the observed values for well-nourished, school-aged Indian children, which was 33.5 mg/kg per day [12]. However, the lysine requirement of undernourished Indian children with intestinal parasitic infestation was observed to increase by 20 %, reaching a level of 42.8 mg/kg per day. Interestingly, the administration of antiparasitic treatments successfully decreased the requirement to 35.5 mg/kg per day [19], making it comparable with the values given by Elango et al. [11] and Pillai et al. [12].

It is evident that some variation in the estimated values can be seen when comparing the outcomes of different studies. The variations are likely due to the differences in the study design (age of the subjects and the size of the study group) and the methodology used for the estimation. Further, the occurrence of subclinical infections (unremarkable in the studies) can also affect individuals' lysine requirement. Therefore, it is essential to pay attention to the dietary requirement of lysine and investigate if the requirement is met through the diet when implementing lysine supplementation.

### Lysine supplements for enhancing the protein quality of the diet

Protein quality pertains to the ability of dietary protein to meet the body's essential needs for regular metabolism and the maintenance or growth of body tissues [20]. The limited presence of lysine in cereal-derived diets has a notable impact on the overall protein quality of the diet [3], [7]. Therefore, the external addition of lysine is necessary to provide a high-quality diet for children, ensuring the proper functioning and development of their bodies. Numerous studies have suggested that supplementing cereal diets primarily with lysine and other amino acids can significantly enhance their protein and nutritional quality, making it comparable to that of a standard diet.

According to a rat study, sufficient lysine added to a bread diet resulted in a significant increase in growth rate, particularly with higher levels of gluten in the diet. The presence of lysine, therefore, plays a crucial role in enhancing the nutritive value of bread protein [21]. Moreover, the addition of lysine to an improved corn meal-based diet (70 % calorie-restricted, with additional micronutrients and soy protein) was found to enhance the overall effectiveness of the diet [22]. An attempt was made to evaluate the linear programming techniques in formulating human diets using rat-feeding tests. The study highlighted that supplementation of wheat with lysine and possibly methionine would meet the protein, amino acids, and energy requirements of a preschool child's diet. However, supplementing rice with lysine alone would not be sufficient to fulfill the child's dietary requirements [23].

Bressani et al. [24] compared the pattern of essential amino acids in the wheat basal diet with the Food and Agriculture (FAO) “reference (egg) protein.” They stated that lysine is the most limiting amino acid in the wheat diet. However, Bressani further described that lysine-supplemented diets could possess similar nitrogen retention as indicated in milk feedings or in basal wheat diets supplemented to resemble the FAO “reference protein” pattern (in the order of lysine, tryptophan, methionine, isoleucine, valine, and threonine). In fact, the combination of lysine and tryptophan supplementation in basal wheat diets showed comparable or even higher nitrogen retention as feeding isonitrogenous quantities of milk [24].

Similarly, lysine and threonine-supplemented partially hydrolyzed rice-protein-based infant formula was also found to replicate the standard intact milk protein-based formula values for growth, plasma biochemistry, and tolerance of 38–42-week-old infants. In this instance, the fortification of lysine and threonine improved the nutritional quality of the rice formula in supporting the expected growth of infants, thereby ameliorating the restricted usability of rice as a primary protein source in the diet [25]. Refined wheat protein supplemented with lysine and potassium exerted almost similar nitrogen retention as found in milk, and the modified diet was suggested as an adequate protein source for infants within tested periods [26].

When examining the results of various clinical studies, there are divergent opinions concerning the necessity of lysine supplements in cereal diets. According to Reddy [27], supplementation of lysine might not be necessary if children are consuming a wheat-based diet that supplies proteins with adequate contents. Nevertheless, Graham and co-workers [28], [29] declared the inability of unsupplemented wheat flour to fulfill the protein requirement of infants and a majority of small children. Graham's team emphasized the significance of adding lysine to the wheat diet for infants, as it resulted in a notable increase in both weight and nitrogen retention.

When going through the findings of overall studies, it is clear that the addition of lysine to a cereal diet can improve its protein quality, and the effect of lysine on growth can be enhanced when it is administered together with other nutrients. These findings hold significant importance in improving the nutritional quality of the diet, particularly in individuals in developing countries who rely on cereals as staples.

### Lysine supplementation for physiological parameters and growth-related functions of animal models

Many research endeavors have investigated how the administration of lysine can potentially alter and modify various physiological and growth-related parameters in rats. These animal studies provide a foundation to understand the potential implications of lysine supplements on biological and physiological parameters, offering a broader perspective on the human application of lysine supplements.

A study carried out by Jing & Li [30] showed that higher dietary lysine levels were responsible for increasing the relative abundance of IGF-I mRNA in rat muscles and liver. However, the higher lysine levels did not affect the serum concentrations of growth hormone, total protein, glucose, insulin, and glucose. When the dietary lysine content of the growing rat's diet was increased within the range of 5.5–9.5 g/kg of the diet, both serum IGF-1 levels and urea nitrogen exhibited a quadratic increase. Supplementation of corn meal diet with lysine accounted for a significantly increased body length. It also elevated the plasma levels of albumin, IGF-1, and other essential amino acids (leucine, isoleucine, and valine) in malnourished young male rats [22]. However, according to Pallaro et al. [31], the administration of lysine (8.38 g/100 g of protein) to a 6.5 % protein corn diet did not exert a sufficient influence on the body weight of growing rats.

Lysine supplements were found to have an important role in inhibiting protein degradation and stimulating protein synthesis in the skeletal muscle of growing rats [32], [33]. According to a study, receiving 22.8–570 mg lysine per 100 g of body weight (bw) was able to inhibit the degradation of myofibrillar proteins in rats. This effect possibly occurs through the autophagy-lysosomal pathway rather than the ubiquitin-proteasomal pathway. Regardless of the degradation pathway, no other impact on protein synthesis of skeletal muscle was found [33]. However, according to Yang et al. [32], partially protein-depleted rats possessed the ability to restore tissue protein levels when fed a delayed lysine supplement. The rats were fed an otherwise adequate but lysine-free diet for 12 h daily, followed by a 12-hour period during which they were given a protein-free diet supplemented with lysine. The effectiveness of the delayed lysine supplement was attributed to the slow turnover rate of lysine in the body or the rats' ability to reutilize some of the lysine (acquired from the breakdown of tissue proteins) for the synthesis of new proteins or a combination of both factors [32].

Lysine supplements are essential for cellular proliferation and maturation of the thymus in growing rats. At weaning, rats were fed a 6.5 % protein corn diet supplemented with lysine at a rate of 8.38 g/100 g of protein to study this aspect. Interestingly, the administration of the supplemented diet reversed the thymic atrophy caused by consuming the unsupplemented cereal diet [31].

Lysine serves as a precursor for carnitine; insufficient lysine intake can result in a deficiency of carnitine in the body. Feeding of weanling rats with an unsupplemented wheat diet (offering roughly 5 % protein derived from wheat) for 10 weeks exhibited severe impairments in growth. It lowered carnitine concentrations in skeletal muscle and plasma. Further, these rats exhibited impaired fatty acid oxidation in the heart, markedly increasing the triglyceride loads in skeletal muscle, heart, and liver. Supplementing the wheat diet with lysine (0.2 %) together with threonine was effective in mitigating the negative impact of carnitine deficiency induced by the lack of dietary lysine in rats [34].

Deficiencies in essential amino acids (such as lysine) in children can expose them to a risk of growth retardation. However, the efficacy of supplementation in recovering deficiency-derived growth retardation is questionable. A study conducted to investigate this scenario by Roisné-Hamelin et al. [35] compared the body parameters of growing rats who were fed a lysine-deficient diet for three weeks, followed by a lysine supplement. The body weight, lean body mass, and naso-anal length of the rats were found to decrease upon receiving the lysine-deficit diet. Although improvements in these parameters occurred after receiving the supplement, it was not effective in overcoming the growth retardation induced by the deficiency.

Overall, these animal studies provide an overview of the usability of lysine supplements in augmenting standard diets and improving physiological functions. Many research findings proved that lysine supplements could reverse the negative impact caused by lysine or protein deficiency. However, the results of Roisné-Hamelin et al. [35] raised a concern regarding the employability of lysine in improving the growth of children and adolescents who were in a state of lysine deficiency. Therefore, the investigation of the nutritional status of children and adolescents and determining the appropriate doses and duration is necessary to ensure the effect on improving growth retardation.

## Lysine supplement for the growth of children and adolescents

Food supplements are widely used to overcome many nutritional problems associated with children and adolescents. The use of lysine-rich or lysine-fortified food supplementation has a positive influence on growth and can ameliorate growth-related health conditions. Indeed, a substantial number of clinical studies have been conducted to investigate this topic. In line with identifying the effectiveness of supplementation of the (primary) cereal dietary staple, a significant proportion of the clinical research has been conducted on populations in developing countries.

### The impact on nitrogen balance

Nitrogen balance is indicated by the net continuous protein anabolism and catabolism in the body. It is influenced by both protein intake and the quality of the dietary proteins [36]. Numerous research findings support the association between lysine supplements and a positive nitrogen balance in the body. For instance, the addition of lysine to the basal wheat diet was found to increase nitrogen retention in children notably. In contrast, the combined supplementation of lysine and tryptophan exhibited similar or higher nitrogen retention than the value obtained from feeding isonitrogenous quantities of milk [24]. In further support, the nitrogen balance of young children was increased notably with the addition of lysine (0.3 %) together with tryptophan to the corn basal diet [37]. These findings align with a previous study [38], which demonstrated that the simultaneous addition of lysine and tryptophan to a corn-based diet significantly increased nitrogen retention in young children who had recently recovered from Kwashiorkor.

Meanwhile, young children who received rolled oats diets containing 308 mg/g N of lysine (at an intake of 1.5 g of protein/kg bw) exhibited a certain degree of improvement in nitrogen retention. However, the observed improvement was only significant when threonine was also supplemented alongside lysine [39]. The output of this research series emphasized the importance of balancing the lysine content and optimizing other amino acids in the supplemented diet. Thus, the amino acid composition of the diet should be taken into consideration when incorporating amino acid supplements to achieve the intended benefits of the dietary intervention.

Lysine supplementation in wheat flour exhibited a beneficial influence on the nitrogen utilization of young men regardless of their energy intake patterns. The measurement analysis of late-adolescents who consumed wheat flour supplemented with lysine (at 2.25 % of the total protein intake) rendered an improved nitrogen balance in the subjects, both under conditions of adequate and restricted calorie intake [40]. This indicates that the lysine supplement has the potential to enhance the utilization of wheat protein in individuals who are experiencing a loss of body weight due to insufficient energy intake.

Several studies have provided some conflicting results regarding the correlation between lysine and nitrogen retention. For example, the supplementation of a corn basal diet with 0.30 % lysine (180 mg/g N) and 0.28 % tryptophan (75 mg/g N) was found to double the nitrogen retention. However, increasing the lysine supplement level to 0.56 % (270 mg/g N) or tryptophan level to 0.35 % (90 mg/g N), either individually or together, did not find a significant increase in nitrogen balance compared to the outcomes observed with these amino acids at lower supplementation levels [37]. Similarly, fortification of wheat with lysine monohydrochloride at 0.1 % levels was also not found effective in increasing the nitrogen balance significantly [27]. The supplementation of the corn diet with different combinations of lysine, tryptophan, and isoleucine was not as effective in improving the nitrogen balance compared to feeding isonitrogenous levels of milk [37]. Meanwhile, children who received wheat protein supplemented with various levels of lysine did not exhibit any significant difference in nitrogen retention [41].

Hence, these studies showed that increasing lysine content may not always lead to a significant increment in nitrogen retention. Additionally, a number of factors may contribute to contradicting results in research, including the variations in experimental design and the study populations. For instance, the size of the population, duration of the study, methods of data collection, level and duration of lysine supplementation, dietary composition of the basal food, and physiological and genetic factors of the population may influence the observations of a study. Hence, novel and in-depth studies on this aspect are recommended to explore more knowledge on this aspect.

In general, the body of evidence confirmed that lysine supplements have the potential to increase nitrogen retention in children and adolescents. However, more studies on supplemental and dietary conditions to identify the optimal level of supplementation are required to achieve a desirable level of nitrogen retention. Investigating how the levels of other limiting amino acids impact the nitrogen retention ability of lysine is essential, as its effectiveness seems to vary depending on the presence of other amino acids. Subsequently, it is essential to consider not only the optimum levels of lysine and other amino acids but also the range within which these levels may fluctuate.

### Effect on growth and health

Studies to assess lysine supplementation in cereal-based staple foods clarify the amino acid's effect on growth and health. The promising impact observed on both healthy individuals and those with minor health conditions (excluding existing malnutrition or in recovery) is reviewed to gain a better understanding of the effects of supplements on the general populations of children and adolescents.

**Fig. 2:**
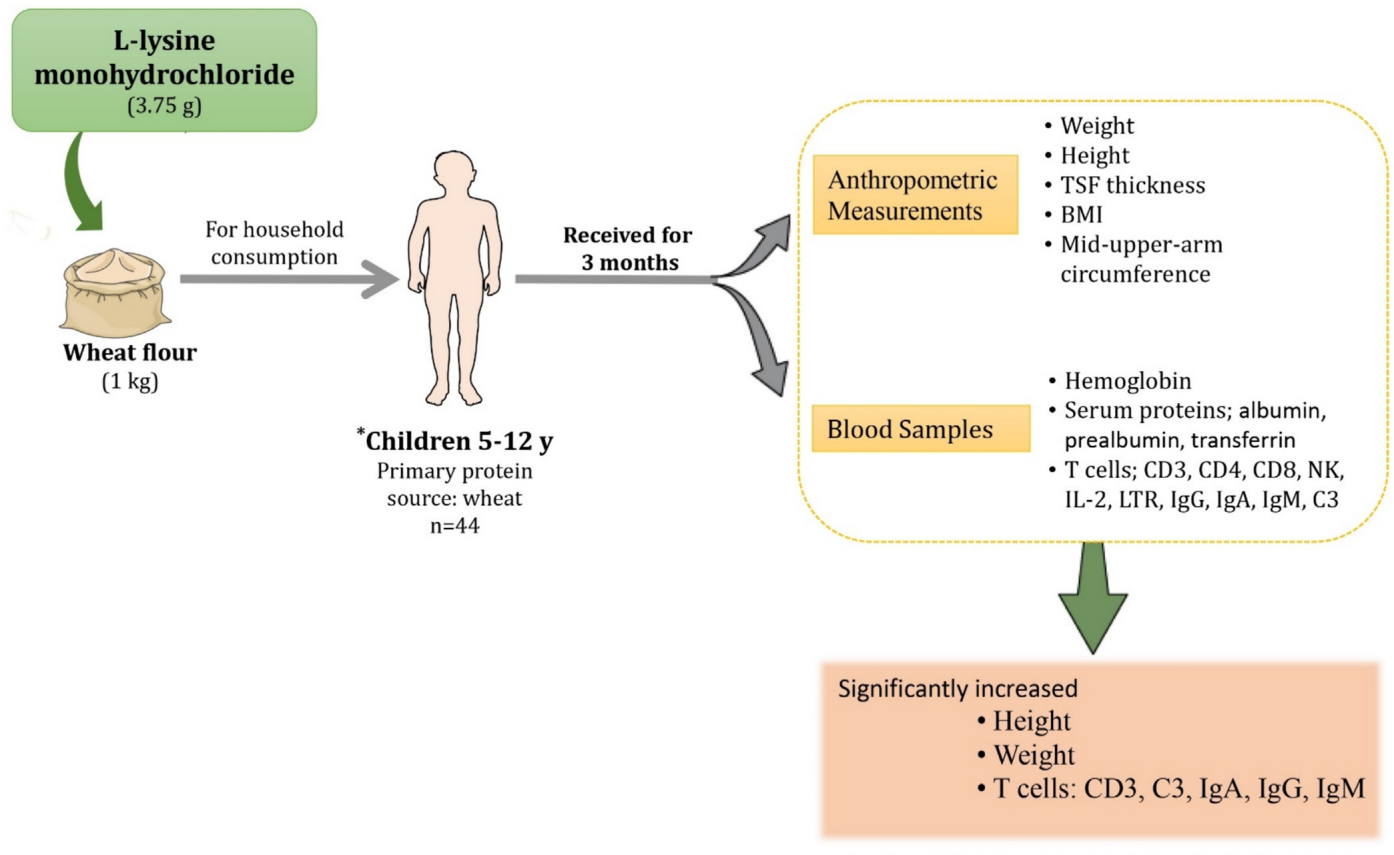
The promising effect of lysine-supplemented wheat products on *children. Abbreviations: TSF thickness, triceps skinfold thickness; BMI, body mass index; IgA, immunoglobulin A; IgG, immunoglobulin G; IgM immunoglobulin M. *5–12 years old children from farm families in China, consumed a diet where 58 %–67 % of their dietary protein intake was sourced from wheat. The experimental group that received lysine-supplemented wheat flour (3.75 g of l-lysine monohydrochloride per 1 kg of flour) for three months exhibited a significant increase in their height, weight, and some immunological indicators [43].

Children in Pakistan who consumed wheat-based products for their main diets were selected and fed with lysine-fortified wheat flour to find the efficacy of lysine on their growth. The consumption of lysine-fortified wheat not only led to significant improvements in weight and height but also positively improved the immunological indicators in the children [42]. Similar findings on nutritional and immune function indicators were reported by Zhao et al. [43] based on a study conducted on 5–12 years old children of farming families in China who were fed with lysine-fortified wheat flour (Fig. 2). However, further studies to identify the underlying mechanism of lysine supplements in improving immune function would be more insightful. It may reveal more aspects of lysine supplements related to cognitive development and functions.

Meanwhile, Ghosh et al. [44] explained that administering lysine supplements for 16 weeks reduced the incidence of diarrhea morbidity among children in Ghana. Lysine supported weight gain by diminishing problematic weight loss resulting from diarrhea. Diarrhea morbidity can be identified as a concern among children in some developing countries. Therefore, the ability of lysine to mitigate the negative impact of health conditions like diarrhea makes lysine supplements a significant nutrition intervention, especially in vulnerable populations.

A study conducted on Japanese elementary school children affirmed that the consumption of lysine hydrochloride-supplemented bread leads to increased body weight, height, and sitting length in children 6–9 years old. Further, the use of lysine supplements along with l-tryptophan, threonine, and l-methionine accounted for a significant improvement in their growth [45]. Pereira et al. [46] stated that incorporating an additional 0.37 g/day of lysine into a wheat-based diet could unequivocally improve the growth of healthy preschool children aged 2–5 years. Meanwhile, Mack et al. [47] studied how lysine supplements are associated with the development of skeletal density in children of preadolescent age. Modification of the tryptophan ratio from 5:1 to 7.9:1 was accomplished by incorporating lysine supplements. The children who received the supplements presented significantly improved weight and height development and yielded a significantly higher mean density of the axial section of the radius. The reported studies provided substantial evidence to explain the fact that modification of the amino acid composition of the diet can play a significant role in enhancing the intended benefits of lysine supplementation on growth.

However, contrary to these anabolic-indicative findings, the administration of lysine-supplemented wheat for 16 weeks did not yield any significant impact on the anthropometric or biochemical parameters of children. Suggestively, the low baseline of diarrhoeal disease rates at the time of receiving supplements could be the reason for the lack of noticeable results in this cohort [48]. A similar incident in Thailand was reported by Gershoff et al. [49], who studied the effect of rice fortified with lysine, other additives (threonine, riboflavin, thiamine, vitamin A), and iron on preschool children. After a 4-year study period, the consumption of fortified rice two-thirds or more of the time did not cause a measurable impact when compared with the placebo group. This non-significant result against placebo was also noted against a comparison population who ate the fortified rice 10 % or less of the time. The results of these studies explained the importance of identifying the complexity of nutritional intervention assignments that can affect the outcome. Therefore, it is clear that there is a need for more thorough context-specific studies using robust methodologies employing different populations.

### Effect on growth and health of malnourished or previously malnourished individuals

The administration of dietary lysine supplements has a promising effect on the recovery and growth of malnourished children. The response to lysine requirement in malnourished children may vary compared to healthy subjects, owing to their altered dietary needs. A number of studies have been done targeting malnourished children, particularly in developing countries. Previously, severely malnourished infants consuming lysine-supplemented (0.12 %) wheat were found to experience increases in weight gain rate, nitrogen retention, and plasma lysine content. These findings prompted recommendations for white wheat flour fortification with lysine at 0.12 % and possibly 0.2 % for infants and children who get cereal-derived proteins as the primary protein source [50].

According to King et al. [51], schoolchildren who were chronically undernourished were provided with bread supplemented with lysine monohydrochloride for an entire school year. The children reportedly experienced an improvement in weight, stature, skinfold thickness, and mean corpuscular hemoglobin concentration [51]. In separate findings, a prolonged supplementation of lysine and threonine indicated a promising effect in enhancing the cognitive abilities of malnourished children. After receiving supplements for three years, monozygotic twins (2–3 years old) diagnosed with mild to moderate protein-calorie malnutrition exhibited significant growth in cognition [52].

Contradictory results were observed in children who were in the process of recovering from Kwashiorkor in the setting of undernutrition conditions. Herein, children were fed lysine-fortified rice and a milk diet. Thus, no difference was found in either the growth rate or level of plasma proteins. The study explained that lysine levels of 115–200 mg/day/kg bw in a diet providing 1.9–3.4 g/day/kg bw of proteins and 46–85 per day/kg bw of calories were ineffective in promoting the recovery of children [53].

Studies elaborating on the promising effects of lysine supplements for infants with nutrition-related issues point towards physiological and anthropometric recoveries. A group of previously malnourished infants aged between 3–8 months exhibited positive outcomes after receiving lysine supplements. The infants were provided quality-improved wheat protein with incremental supplementation (from 0.12 % to 0.4 %) with lysine. The rate of weight gain was found to improve with the addition of lysine to the diet [41]. Meanwhile, the impact of lysine on correcting the indicators of protein inadequacy was elaborated using a study group consisting of infants and children who were recovering from malnutrition. Interestingly, withdrawal of lysine supplement was not found to affect the tested protein adequacy indicators [28], [29].

Hence, these findings suggested that lysine supplements can be beneficial in improving the development and cognitive function of children and infants who are at different stages of malnourishment or previously malnourished conditions. Despite the reported benefits, the research outcomes of Wikramanayake et al. [53] helped to understand that lysine supplementation might not be employable as a standard therapy for all malnourished subjects. Therefore, it is important to note that the impact of lysine on malnourished conditions could vary for many possible underlying reasons. For instance, the methodology employed (supplement dosage, type of basal diet, duration) and the status of the study population (age, severity of malnutrition, stage of recovery, other health conditions, concurrent treatments) can impact the results of the study.

### Effect on individuals with short stature

Stunting among children has become a global concern as approximately one-quarter of children in the world who are below five years of age are reportedly affected by stunting. Impaired nutritional intake is a predominant cause of this condition, with insufficient lysine intake believed to increase the risk of stunting [54]. Idiopathic short stature (ISS) is the most prevalent short-stature type in children, and it holds 60–80 % of all short-stature children. Interestingly, lysine supplements were found to play a significant role in promoting height achievement in short-stature children.

**Fig. 3:**
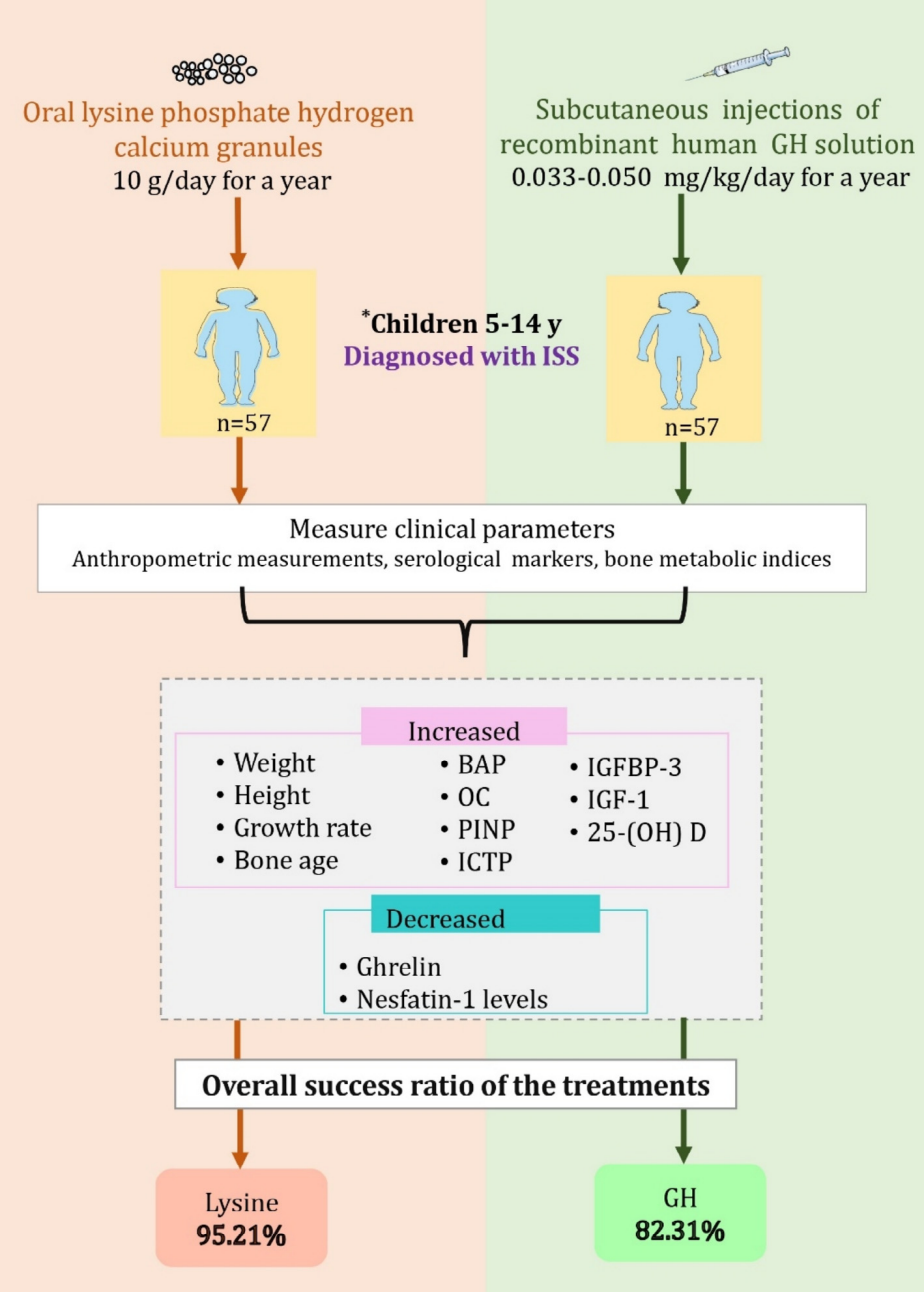
Promising effect of lysine oral supplements on *children diagnosed with idiopathic short stature. Abbreviations: ISS; idiopathic short stature; GHBP, growth hormone-binding protein; GHRH, Growth hormone-releasing hormone; IGF-1, insulin-like growth factor-1; nesfatin-1, feeding inhibitory factor-1; GH, growth hormone; IGFBP-3, insulin-like growth factor binding protein-3; ghrelin, growth hormone releasing peptide; 25-(OH) D, 25-hydroxyvitamin D; BAP, bone-specific alkaline phosphatase; OC, osteocalcin; PINP, type I procollagen amino-terminal peptide; ICTP, type I collagen cross-linked carboxy-terminal peptide. *5–14 years old children in Iraq diagnosed with ISS and absence of hypercalcemia, drug allergies, mental disorders, sarcoidosis, acute respiratory failure, severe systemic symptoms, complete closure of the epiphysis, or active malignant tumour. The treatment group that received the lysine supplement exerted a significantly greater improvement on the tested parameters compared to those receiving the growth hormone [56].

Xu et al. [55] concluded that an adequate supply of lysine performs an essential role in the treatment approaches for short stature in children. Mohammed et al. [56] also stated the efficacy of receiving oral lysine phosphate hydrogen calcium granules in improving the development rate of weight, height, bone age and growth, bone metabolism indices, and serological parameters (Fig. 3). In fact, the study demonstrated a greater success ratio for the lysine phosphate hydrogen calcium granules compared to recombinant human growth hormone. From these findings, combined therapy of lysine phosphate hydrogen calcium granules with recombinant hormone was stated as a promising method for achieving effective results on ISS children.

The implementation of lysine-inositol vitamin B12 therapy, together with regular and moderate stretching exercises, was found to exhibit a promising effect on the height growth of children with ISS. In this study, the group of children who received the combined therapy showed a significantly improved impact on growth rate when compared with the children who only consumed the supplement [57]. According to these research findings, it is evident that lysine holds significant importance in different therapeutic interventions to improve short stature in children. A combination of lysine supplements with other applicable strategies might, therefore, enhance the overall impact of the prescribed therapy.

### Lysine-rich food sources as promising supplements

There are lysine-rich food sources and plant materials with potential uses as supplements to ameliorate the negative effect of lysine-lacking diets. Previously, a ragi (*Eleusine coracana*, a millet) diet was supplemented to provide 0.05 g of additional lysine per day by using either lysine monohydrochloride or Lucerne leaf protein as the lysine source. The addition of lysine was found to increase height, weight, and nitrogen retention in children despite their source [58]. Moreover, in Indonesia, eel (high lysine and arginine) biscuits were reported to have a beneficial effect on 36–60-month-old children diagnosed with stunting. The consumption of the eel biscuits for three months resulted in an elevated height-for-age z-score in stunted children, suggesting a potent lysine-inclusive factor that is responsible for improved children's growth [59]. These studies explained the employability of natural lysine-rich sources as supplements to promote the growth of children. Therefore, exploration and effective use of novel natural lysine-rich sources could offer a holistic approach to addressing lysine deficiency while providing a diverse nutritional profile to children.

A selection of clinical studies with prominent results are summarized in Table 2. It is worth noting that the majority of these available studies on lysine supplementation for the growth and development of children and adolescents were conducted before the year 2000. Since then, globalization has had a significant impact on socioeconomics and dietary patterns worldwide. Therefore, conducting more contemporary clinical studies in this regard would provide more insightful and up-to-date information.

**Table 2:** Effect of lysine supplement on the growth of children and adolescents: Highlights of clinical studies.

Characteristics of the sample	Nutritional Status/diet of the study population (prior supplement)	Study plan	Duration of supplement	Measured factors/parameters	Impact of the supplement	Reference
Monozygotic twins (2 to 3 years old) in the Tohoku district in Japan Total sample size: 6 pairs	Children had low protein and fat intake. Rice was their dietary staple, with eggs and fish in small quantities only 2 times per week.	One child of each twin pair received a tablet of 0.1 g of lysine and a tablet of 0.05 g of threonine; the other child received placebo tablets.	1,300 days	D.Q, I.Q	A significant rise in D.Q was observed, and the rise was notable in the early phases of receiving the supplement. The impact on I.Q. was unclear in both experimental and control groups.	[52]
Infants (3–17 months old) in Latin-America Total sample size: 22	Hospitalized children due to severe diarrhea and had been kept the condition under control using usual treatments. They were fed with milk and good-quality protein for at least 10 days before the experiment.	Each infant was fed a diet containing lysine monohydrochloride at a total amount of 0.55 % wheat and potassium. The infant received 75–120 cal/kg of calories and 1.2–4 g/kg of proteins per day from the diet.	27–54 days (4–5 times daily)	Weight, urine, faces, blood measurements	Increase nitrogen retention when having adequate potassium in the diet.	[26]
Infants (3–8 months old)Total sample size: 6	Previously malnourished children who are staying in the hospital for a considerable period to ensure their gain of weight and linear growth	Each infant was fed with wheat diets that contained 0.12, 0.2, and 0.4 % lysine.	15 to 36 days	Weight gain rate, nitrogen retention	The growth rate increased with the addition of lysine. No significant impact was found on the estimated nitrogen percentage in the gained weight.	[41]
Children (2–12 years) old in GhanaTotal sample size: 45	Children from different households in *peri*-urban regions who had not been diagnosed with serious illness or anemia were included in the study.	The test group was provided with lysine hydrochloride tablets (total of 1000 mg lysine), while the placebo group received tablets of dibasic calcium phosphate (1200 mg).	16 weeks (daily)	Health status, weight, height, the venous blood sample	The treatment significantly diminished diarrheal morbidity and the impact of diarrhea on weight gain without significantly affecting the biochemical measures.	[44]
Children (6–12 years) in India.Total sample size: 80	Children did not have apparent diseases or abnormal clinical symptoms.	Children were grouped, and one group was fed with a ragi diet supplemented with 0.66 g of lysine monohydrochloride. The other group was fed a ragi diet supplemented with 15 g of lucerne leaf protein. Both supplemented diets contained 0.5 g of lysine.	6 months (daily)	Weight, height, red blood cell count, hemoglobin, nitrogen balance	Increased growth, nitrogen retention, red blood cell count, and hemoglobin content were observed.	[58]
Children (5–10 years) in Pakistan.Total sample size: 80	Around 56 % of the targeted group were found to be wasted or stunted. Marasmus or kwashiorkor incidents were not reported, but listlessness and fatigue were commonly seen among children, while 68 % were found to have parasitic infections.	Children in the test group were provided with wheat flour fortified with lysine at 0.6 g lysine/100 g. The control group received unfortified flour. Wheat source provided 58 % of protein. Flour was distributed to the households according to their needs, and the groups consumed only this flour during the period.	6 months. (individual intake was not monitored)	Weight, height, TSF thickness, BMI, prealbumin, transferrin, T-cell populations (CD4, CD8, complement C3), hemoglobin	Treatment significantly increased the weight, height, transferrin levels, CD8, CD4, and complement C3 but reduced prealbumin levels. There were no significant changes in BMI, TSF thickness, and hemoglobin levels.	[42]
Children in ChinaTotal sample size:60	Children were diagnosed with ISS	Both the control and observation groups received 10 ml lysine-inositol vitamin B12 oral solution. Only the observation group exercised according to the ISS exercise sheet.	12 months (twice per day)	Height, height standard deviation score, growth velocity, biochemical indicators	The combined therapy with exercise significantly increased height, growth velocity, and GHBP, GHRH, IGF-1, GH, and IGFBP-3 levels in serum while significantly lowering the height standard deviation score. No significant changes were found in the biochemical indicators.	[57]
Sub-adolescent children (6–12 years) old in AmericaTotal sample size: 84	Children were found to consume common basic diets.	The test group was fed a lysine-supplemented diet (lysine-tryptophane ratio of 7.9:1), while the control group received a basic diet (lysine-tryptophane ratio of 5:1).	5 and a half months	Growth, skeletal density	Treatment significantly improved the weight and height development while resulting in a significantly higher mean gain in the density of the axial section of the radius. There was no significant mean gain in os calcis density.	[47]
Preschool children (2–5 years old) in IndiaTotal sample size: 52	The children were residents in an orphanage. They were in good health, and minor illnesses were maintained during the period. Their diet was maintained (supplied 2 g vegetable protein, 100 kcal/kg bw per day) for 3 months before the study.	The experimental group was fed a lysine-supplemented wheat diet that supplied 2 g vegetable protein, 100 kcal/kg bw per day. Wheat in the diet provided 85 % of the daily protein and 54 % of the daily calories. The wheat flour and the broken wheat used to prepare the diet were added with lysine monohydrochloride (to provide 0.25 % of supplements) and lysine-impregnated wheat grain (to provide 0.1 % of supplements), respectively.	6 months	Height, weight, hemoglobin, total serum proteins, nitrogen balance	A significantly increased height was observed. There were no significant differences in weight gain, nitrogen retention, packed cell volume, hemoglobin, serum albumin, and serum protein.	[46]
School children (4–18 years old) in the Republic of Haiti.Total sample size:464	Children were chronically undernourished and lived in rural villages.	The experimental group received 150 g of lysine-supplemented bread with 9 g of guava jelly per day. The bread was supplemented with lysine hydrochloride at the level of 625 mg/100 g of flour.	One complete school year	Height, weight, skinfold thickness, clinical parameters, hemoglobin, hematocrit values, protein fractions, and total proteins in serum	Increased weight, stature, corpuscular hemoglobin concentration, and skinfold thickness were observed. There was no significant impact on serum total protein, serum albumin, and cholesterol levels.	[51]
Infants (11–24 months old)Total sample size:6	Previously severely malnourished children who had been treated until observing a steady weight gain rate, corrected hepatic steatosis, and normal serum protein level.	Infants were fed with a diet made from processed white wheat flour (contained ∼ 21 % protein) supplemented with lysine (lysine hydrochloride) at different levels. Supplement levels were equivalent to 0.12 %, 0.2 %, and 0.4 % lysine-enrichment of ordinary white flour.	15–36 days	Rate of weight gain, nitrogen balances, serum albumins, total proteins, fasting plasma amino acids	Supplementation at 12 % improved the weight gain rate and nitrogen retention. It also increased the plasma lysine molar ratio and stabilized the serum albumin. Supplementation at 0.2 % level further improved the rate of weight gain and nitrogen retention. Further, it resulted in increased lysine levels and decreased threonine levels in plasma. Supplementation at 0.4 % suggestively further elevated nitrogen retention and resulted in increased lysine levels and decreased threonine levels in plasma.	[50]
Young men (18–24 years old) Total sample size: 16	The students were in good health.	Young men were fed a diet that included wheat gluten as the primary source of protein. Daily protein intake of experimental groups 1 and 2 were 0.27 and 0.73 g per kg bw, respectively. The diet was supplemented with lysine at 2.25 % of the total protein intake. The diet lasted for 60 days in two 30-day periods; in one period, adequate calories were received, and in the other period, calorie intake was 20 % less.	15 days in each period	Nitrogen balance, fasting, and postprandial total protein, albumin, and urea levels in the serum	Treatment significantly increased the nitrogen balance in both restricted and adequate energy levels. Further, it significantly increased albumin at low protein intake. There was no impact on blood urea.	[40]
Children (4–15 years old) in northwest Syria Total sample size: 69	Children were selected from rural families that reside in areas where comparatively higher levels of stunting and underweight incidence were reported.	The experimental group received the lysine-added wheat flour at a rate of 4.2 g of lysine hydrochloride per kg of flour. The control received the unfortified flour.	16 weeks	Weight, height, weight-for-age, height-for-age, weight-for-height z score, BMI, biochemical analysis	There was no apparent impact on anthropometric and biochemical parameters.	[48]
Infants (38–42 weeks) Total sample size: 65	Infants in good health were selected for the study. They were not breastfed by mothers and had no congenital or acquired conditions.	The experimental group fed lysine (4769 µmol lysine/L) and threonine (2518 µmol threonine/L) fortified partially hydrolyzed rice protein-based formula. The control group fed standard intact cow's milk protein-based formula.	16 week	weight, length, head circumference, and biochemical analysis.	There was no impact on the anthropometric parameters. The experimental group resulted in lower plasma levels of urea nitrogen, phosphorous, and essential amino acids (except threonine). There was no difference in total protein, prealbumin, albumin, magnesium, calcium, magnesium, or alkaline phosphatase.	[25]
Infants (6.4–24.5 months old) Total sample size: 6	The children were previously malnourished. They were staying in the hospital and were well along in recovery. They had a steady weight gain with no infections.	Children received a wheat-based (75 %) diet added with 0.2 % of lysine.	3 months (fed with the wheat diet for three months, and lysine was added in the second month only)	Weight gain, nitrogen retention, cholesterol, and albumin levels in serum	Lysine supplements significantly increase weight gain, nitrogen retention, and stabilized albumin levels, whereas the withdrawal of the supplement significantly reduced the weight gain. There was no effect on nitrogen retention or albumin.	[28], [29]
Children (36–60 months old) in IndonesiaTotal sample size: 56	The children had a short or very short height (with a height-for-age z-score of less than − 2 S.D).	The intervention group was fed 10 pieces of eel biscuits (containing 4490.13 mg of lysine per biscuit). The control group received biscuits without the eel formula.	3 months	Height-for-age z-score	Supplemented eel biscuits led to an increasing of height-for-age z-score.	[59]
Children (2–4 years old) Total sample size: 7	Children had a daily protein intake of 2 g/kg and a daily calorie intake of 90–100 kg.	Experiment 1: A child received isoproteic levels of milk and wheat flour supplemented with lysine (270 mg lysine/g of nitrogen in diet). Another child received the same quantity of milk and wheat flour supplemented with other limiting amino acids.Experiment 2: Children received wheat flour added with different amounts of lysine (162 mg −282 mg of lysine/g of nitrogen in diet)	3 days	Nitrogen balance	Nitrogen retention in children was found to increase with the addition of lysine. The maximal nitrogen retention was achieved by supplementing the basal diet with 162 and 194 mg of lysine/ g of nitrogen for daily protein intake rates of 2 and 3 g/ kg of bw, respectively.	[24]

Abbreviations: bw, body weight; D.Q, developmental quotient; I.Q, intelligence quotient; TSF thickness, triceps skinfold thickness; BMI, body mass index; ISS; idiopathic short stature; GHBP, growth hormone-binding protein; GHRH, Growth hormone-releasing hormone; IGF-1, insulin-like growth factor-1; nesfatin-1; feeding inhibitory factor-1; GH, growth hormone; IGFBP-3, insulin-like growth factor binding protein-3; ghrelin, growth hormone releasing peptide.

### Optimum lysine supplementation for maximum growth

Determining the optimum level of lysine supplementation is crucial for achieving ideal body functions and reducing the risk of adverse effects [60]. When going through the literature, several rat models involving young rats were employed to investigate suitable levels of lysine supplementation in diets with different nutrient and micronutrient compositions.

According to a study, receiving a 0.97 % lysine-fortified wheat-based isonitrogenous diet (comprised of 85 % white wheat flour and 4 % lactalbumin) for 28 days resulted in a maximum weight gain in weanling rats. However, when the white wheat diet added different levels of whey protein, the observed maximum weight gain varied depending on the amount of whey protein included in the diet [61].

Amos et al. [62] stated that supplementing sunflower meals with lysine shows a significant, linear, and quadratic response in weight gain, exhibiting a maximum weight gain at 0.34 % level. Meanwhile, a study group of weanling rats received wheat diets (containing 11.6 % wheat gluten) supplemented with graded levels of lysine for 14 days. Weanlings exhibited the maximum weight gain when receiving lysine hydrochloride between 0.64–1.8 % [63]. In both cases, further increasing lysine levels results in a decline in growth.

Kihlberg & Ericson [64] stated that supplementing the rye diet with lysine at 0.25 % (making the total lysine content of the diet 0.70 %) resulted in maximum weight gain, nitrogen efficiency and lower fat percentage in the liver in growing rats. Meanwhile, it was found that 0.2 % and 0.25 % levels of lysine were the optimum levels for achieving maximum weight gain in weanling rats [60]. According to Rosenberg & Culik [65], lysine at 0.10 % was the appropriate dose, as at that point, the balance was maintained between lysine and threonine. The importance of maintaining optimum levels of lysine and threonine in the wheat and barley diets was elaborated by Heger et al. [66]. Herein, the weight gains and net protein utilization observed in weanling rats were found to be maximum when receiving 0.69–0.70 % of total dietary lysine and 0.52–0.55 % of total dietary threonine.

These rat studies provided insights into the importance of maintaining the optimum level of lysine when used in the supplementation process. The maximum weight gain of rats was found to vary with the level of lysine and as well the presence of other proteins and amino acids in the diet. Therefore, it is essential to carefully determine not only the optimum level of lysine supplementation but also to investigate the compositional and nutritional profile of the diet being supplemented. Such thorough examination is necessary to achieve the maximum benefits of the supplementation.

### Limitations and precautions

This review emphasizes the importance of incorporating lysine supplements into the diet to ensure that lysine intake meets dietary requirements regardless of staple and protein sources. However, it is also essential to evaluate and establish safe upper levels to prevent unnecessary and excessive usage of lysine supplements. The addition of lysine in the free, anhydrous, or hydrated forms of sodium, potassium, or hydrochloride forms to food is recognized as safe when it meets the conditions specified by the Code of Federal Regulations. From these specifications, the total amount of lysine present in finished food in both free and combined form should not exceed 6.4 % by total protein weight [67]. Thus, there are limited documentation and research articles that indicate the upper safe levels of lysine supplementation [68].

Despite the extensive utilization of lysine in dietary supplements across numerous nations, safety data regarding the potential risks associated with consuming excessive amounts of lysine are not abundant [68]. However, some animal and clinical studies have been conducted to assess the adverse impact of potential toxicity and to estimate safe levels of lysine supplements. Highlights of relevant research studies are shown in Table 3.

**Table 3:** Adverse effects and potential toxicity related to lysine supplements.

Study Subjects	Supplement doses	Duration	Adverse impact	Remarks	Reference
**Animal Studies**					
Four weeks old rats	The rats were orally administered a standard diet supplemented with 1.25 %, 2.5 %, and 5 % (w/w) lysine.	13 weeks (ad libitum)	No adverse impact was found	NOAEL was estimated at 5.0 %.	[75]
Weanling rats	Rates were fed diets containing casein as a source of protein (5, 10, 20 or 30 %) supplemented with 5 or 10.0 % lysine.	28 days	The 10 % lysine supplements were found to suppress the growth. Growth suppression was high at low casein levels.	10 % lysine did not have a toxic impact.	[76]
Rats	Rates were fed casein diet (7.5, 15 and 30 %) supplemented with 0, 2.5, and 5 % lysine.	2 weeks	The supplement could influence lipid metabolism. The 5 % of lysine supplements resulted in the accumulation of triglycerides in the liver.	The influence of the lysine supplement dose varied with the casein content in the diet.	[77]
Ten weeks old rats	Rates were fed diets containing casein (7 or 20 %) and added with lysine at levels of 1.5, 3, or 6 %.	1 week	The ≥1.5 % lysine supplementation of a diet with low dietary protein (7 % of casein) might cause negative health effects.	The addition of arginine to the diet ameliorated many of the adverse impacts.	[79]
Rats	Rates were fed a casein diet (15 %) with 5 % lysine.	2 weeks	The 5 % lysine increased triglycerides, cholesterols, and total lipids in the liver.	Fatty liver conditions caused by lysine can be prevented by adding arginine.	[78]

**Clinical Studies**					
4–11 months old infants	Infants were fed 60, 120, 240, 480, 720, 960, and 1080 mg of lysine per 8 oz. cow's milk	3 or 4 days	No adverse impact was observed.	Maximum daily intake was reckoned as 5.18 g per day.	[73]
10–14 years old children	Children were given 0.5 ml of 1 M lysine monohydrochloride per kg bw	Single dose	No adverse impact was observed.	_	[74]
18–50 years old healthy subjects	Doses of 0.5, 1.2, 3.0, and 7.5 g of lysine monohydrate were provided per day.	Lysine test meals were infused for 3 sessions.	Diarrhea was observed within 6 h after receiving high doses (7500 mg) of lysine. No adverse effect was occurred at low doses (500–3000 mg).	_	[71]
21–48 healthy subjects	Subjects were provided with a meal added with 1 mmol lysine per kg of lean bw (mean dose-11000 mg of lysine)	Single dose	The majority of the test subjects had mild gastrointestinal upset, and diarrheal episodes were seen in some subjects.	_	[70]
Adults suffering from fluid retention (caused by liver or heart disease)	10–40 g of lysine monohydrochloride per day was given to each adult.	2–5 days	Occasional abdominal cramping and transient diarrhea were observed.	Side effects were relieved at low doses of lysine.	[69]
Children with ISS	Children were given 10 ml (bid) of lysine-inositol VB12 oral solution	12 months	No adverse impact was observed.	The supplement was found to be clinically safe	[57]

Abbreviations: NOAEL, no observed adverse effect level; ISS, idiopathic short stature.

Adverse events linked to lysine supplements in humans primarily consist of subjective gastrointestinal tract symptoms, including stomachache, nausea, and diarrhea [14], [69], [70], [71]. Nevertheless, when it comes to the safety of humans, plenty of studies have provided valuable insight into the appropriate dosage levels and long-term usage. According to Flodin [72], lysine hydrochloride is well-tolerated and safe for humans when used in doses of up to 3.75 g per day. In terms of long-term usage, Flodin explained that a dosage of 6.0 g of lysine hydrochloride per day was deemed safe based on animal and human trials. This fact was further confirmed by Hayamizu et al. [68], stating that the no-observed-adverse-effect level (NOAEL) of lysine is 6000 mg per day, and the lowest observed-adverse-effect level (LOAEL) of lysine is ∼ 7500 mg per day. Additionally, receiving up to 1080 mg of lysine per 8 oz (236 ml) of cow milk was well-tolerated and safe for infants [73]. Kato et al. [74] explained that a single dose of 0.5 ml of 1 M lysine monohydrochloride per kg body weight was not found to cause any adverse impact on children.

Regarding the toxicity and safety of lysine, some rat models have been employed to assess the toxicity levels and lethal dosages of lysine supplements. According to a study, the lethal dosage of intravenous administration of lysine monohydrochloride in young male rats was reported to be 22 mmol/kg bw. Interestingly, they further explained that no lethal dose could be achieved through oral administration [72]. Meanwhile, a toxicity study in which rats were orally administrated lysine supplements ad libitum for 13 weeks estimated that NOAEL for lysine is at 5.0 % in both males and females [75]. Even higher supplement levels in diets, such as 10 % lysine, consumed for 28 days, were not found to have any toxic effect on rats.

Nevertheless, the findings of some animal studies have raised awareness regarding the possible adverse effects of high levels of lysine supplements in the diet. For instance, an incidence of weight loss and reduced gain rate were observed in growing rats fed with high levels of lysine-supplemented meals [60], [61], [62], [65]. It is reported that a lysine level as high as 1.0 % in the flour diet was found to cause adverse impacts on the tested rat model [60]. Meanwhile, the lipid metabolism of rats was reportedly influenced by the excess lysine contents, leading to triglyceride accumulation in the liver [77], [78].

Taking precautions when implementing lysine supplementation can aid in maximizing the intended benefits and mitigate the possible adverse outcomes resulting from improper use. An essential precaution to consider when using lysine as a supplement is the protein content and the amino acid composition of the basal diet that is being supplemented. According to a rat study, the negative impact of excessive lysine on lipid metabolism was observed to depend on the dietary protein content and the amino acid composition of the diet [77], [78]. Xiao et al. [79] further confirmed the possible adverse impacts of high lysine supplements, emphasizing the need for consideration when the diet contains low levels of dietary proteins. This fact was further supported by Friedman & Finot [80], who stated that the adverse effect of 0.75 % lysine on weight gain in mice was alleviated when the bread diet contained high gluten contents of 20 and 25 %. Further, it was reported that the growth suppression of weanlings caused by high lysine doses was further increased when decreasing the casein content of the diet [76].

Another significant consideration regarding lysine supplementation revolves around its potential impact on the chloride content of the food. Since lysine hydrochloride is the commonly used form in dietary supplements, its addition to the diet also results in an increased intake of chloride [14]. Therefore, it could raise some limitations regarding lysine-supplemented foods, especially in individuals who are concerned about chloride intake. However, despite the increase in chloride, Hayamizu et al. [14] found no evidence of hyperchloremic acidosis caused by lysine supplements. The challenge of distinguishing the effects of lysine from the potential impact of chloride highlights the need for further research in this area to elucidate safety considerations better.

It is important to note that lysine supplementation may not be essential for healthy children and adolescents who obtain sufficient amounts of lysine through their diet. Hence, accurately identifying the need for the supplement is crucial prior to initiating lysine supplementation. However, when scrutinizing the findings of previous research, it is evident that the necessity for lysine supplements can be subjective, and it can depend on various factors such as the dietary intake of lysine, age and development stage, diet patterns, overall nutritional status and medical conditions [7], [8], [11], [13], [19]. Therefore, it is advisable to consult a healthcare professional to take these factors into account before starting lysine supplements. Further, it is essential to monitor the lysine levels regularly during the period of supplementation to alleviate possible adverse effects that can be caused by excessive lysine.

Overall, research findings exclaimed that lysine is a safe supplement that can be used to upgrade the nutritional quality of a diet when it is used with appropriate precautions. The perception of its safeness has led to the application of lysine as a dietary supplement worldwide. Nevertheless, further research is recommended to ensure continued safety.

## Conclusion

As lysine is an essential amino acid, it is crucial to obtain adequate amounts from external sources. However, fulfilling the dietary requirement of lysine solely through food may be challenging because many significant food sources contain limited quantities and a fractional loss of total lysine also occurs due to its high reactivity. Insufficient intake of dietary lysine has caused numerous growth-related issues in children and adolescents who predominantly rely on cereal-based diets as their staple food. Hence, the supplementation of cereal (wheat, rice, and maize) diets with lysine is implemented to meet daily dietary needs around the world.

The findings of both animal and clinical studies from the past few decades elaborate on the promising effect of lysine oral supplements and lysine-fortified diets. Such practice of supplementation improves growth by positively influencing nitrogen retention, anthropometric measurements, serological biochemical parameters, red blood cell parameters, and brain performance. Lysine supplements can identified as safe and well-tolerated in children and adolescents, with minimal adverse effects. However, it is crucial to carefully add lysine in optimal quantities to maintain the proper balance of amino acids in the diet.

Overall, lysine supplementation is a vital dietary intervention to ensure the optimum growth of children and adolescents. However, this review has elucidated that the most crucial findings regarding this aspect are based on research conducted several decades ago. While these studies have provided vital information on lysine supplementation in children and adolescents, it is essential to conduct new studies using innovative concepts and technology, especially considering potential changes in the characteristics of target populations over time. This approach would not only provide a more contemporary perspective on lysine supplementation but also contribute to raising awareness of its importance. In contrast, the absence of new references indicated a complacency in this field, where it was assumed that existing literature provided adequate information. However, in reality, the progression of science and technology should facilitate the development of more profound knowledge, which has not been achieved. This concern emerged from the findings of this review.

There remains a knowledge gap concerning the upper level of dietary intake of lysine that requires further investigation. Further exploration of this aspect is necessary to avoid potential adverse effects of lysine supplementation while maximizing its benefits. Furthermore, in-depth and long-term animal and clinical studies are warranted to investigate the safe dosages and the durations of lysine supplementation across various age groups of children and adolescents.

## Compliance with Ethics Requirements

This article does not contain any studies with human or animal subjects.

## Declaration of competing interest

Professor Jun Lu received research funding from Royal Society of New Zealand Catalyst Fund and EZZ Life Science Holdings Pty Ltd, Australia. However, neither Royal Society of New Zealand nor EZZ Life Science play any role in the writing of this review. No restrictions have been placed by funders on the publication of the review. There is no other conflict of interest to declare by all the authors.
